# Supplementary material for: Seminoma and Embryonal Carcinoma Footprints Identified by Analysis of Integrated Genome-Wide Epigenetic and Expression Profiles of Germ Cell Cancer Cell Lines
Source: PLoS One. 2014 Jun 2;9(6):e98330. doi: 10.1371/journal.pone.0098330 (PMC4041891; doi:10.1371/journal.pone.0098330)
Supplement: File S1 — ZIP file containing DMRforPairs output for significant regions. Please start from the html files. (ZIP) [file pone.0098330.s008.zip › figures/12431.pdf]

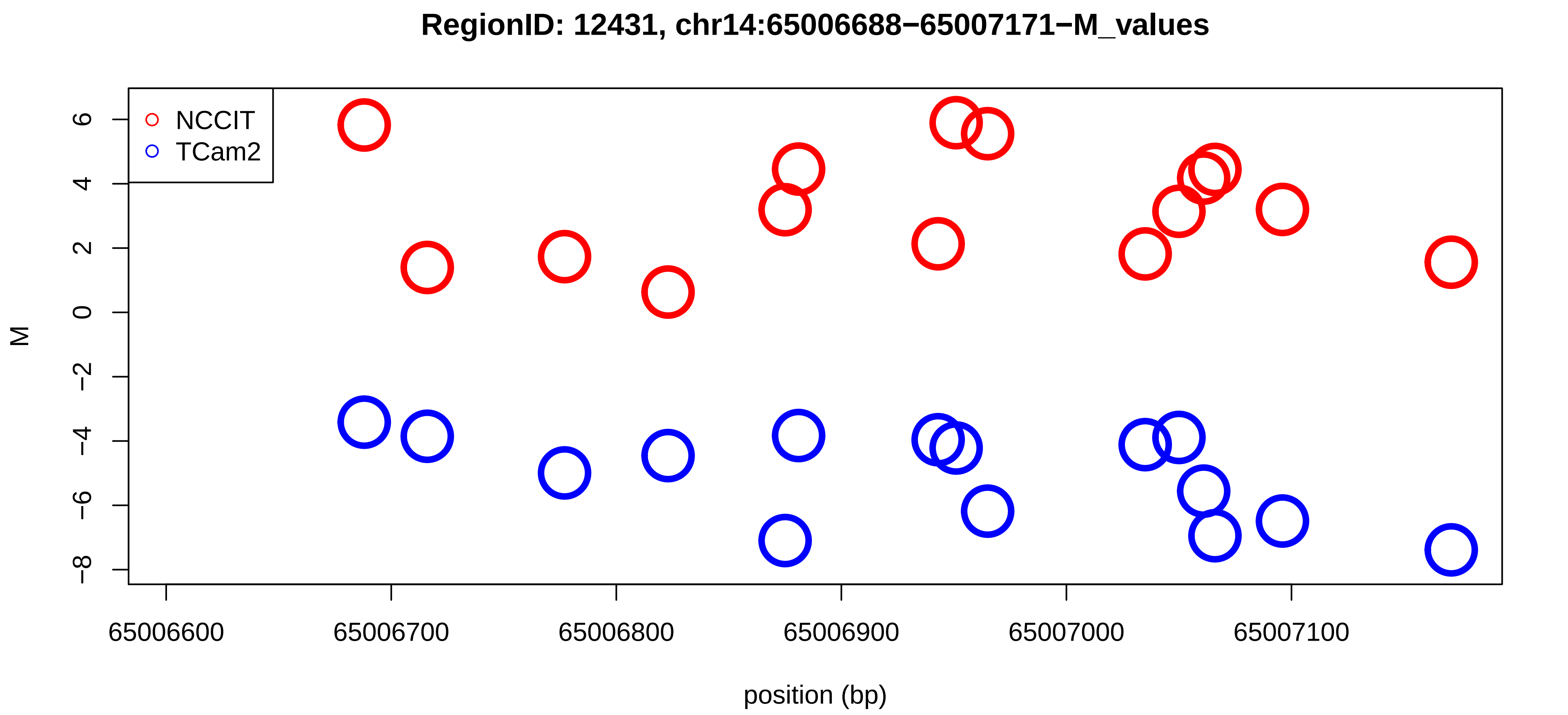

RegionID: 12431, chr14:65006688−65007171−M_values
NCCIT
TCam2
M
6
4
2
0
−2
−4
−6
−8
65006600
65006700
65006800
65006900
65007000
65007100
position (bp)

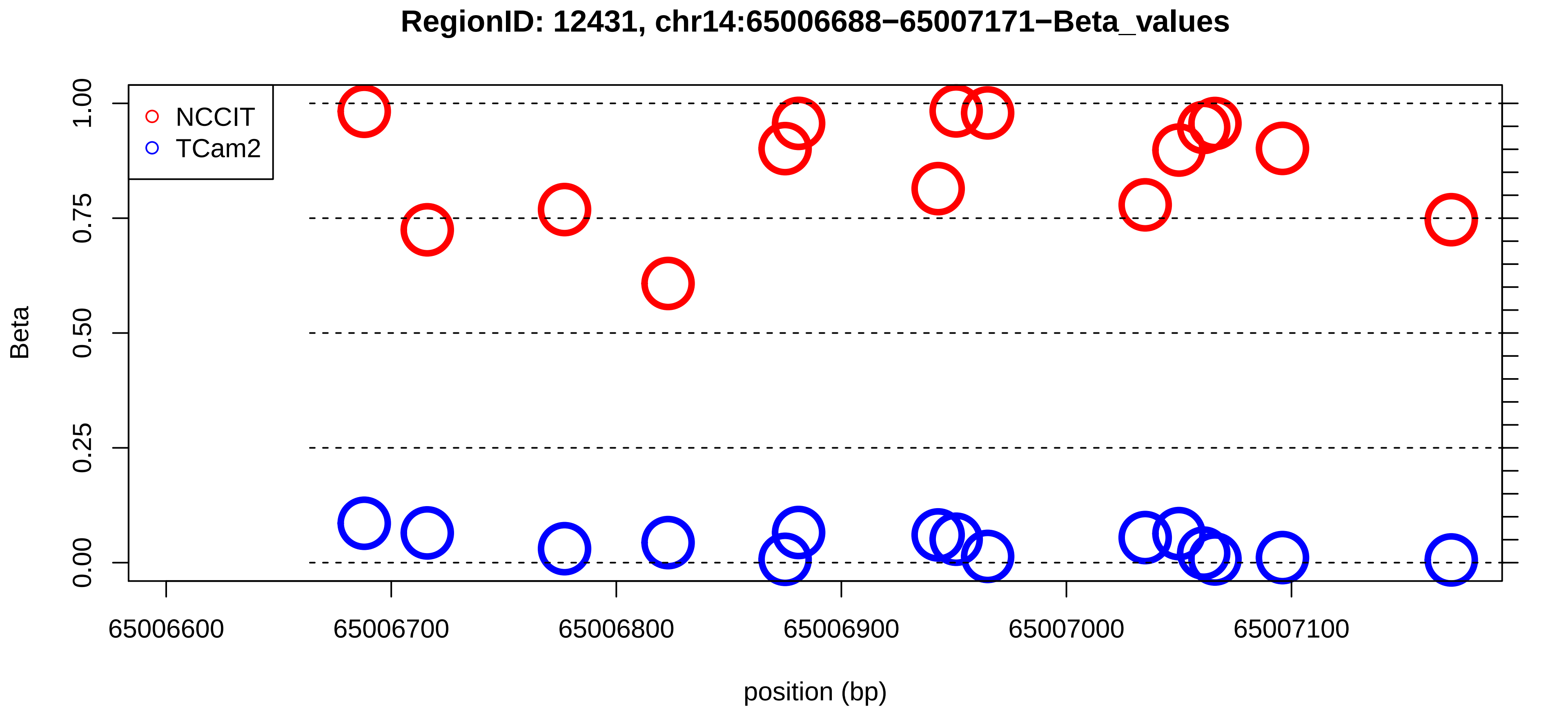

RegionID: 12431, chr14:65006688−65007171−Beta_values
NCCIT
TCam2
Beta
1.00
0.75
0.50
0.25
0.00
65006600
65006700
65006800
65006900
65007000
65007100
position (bp)

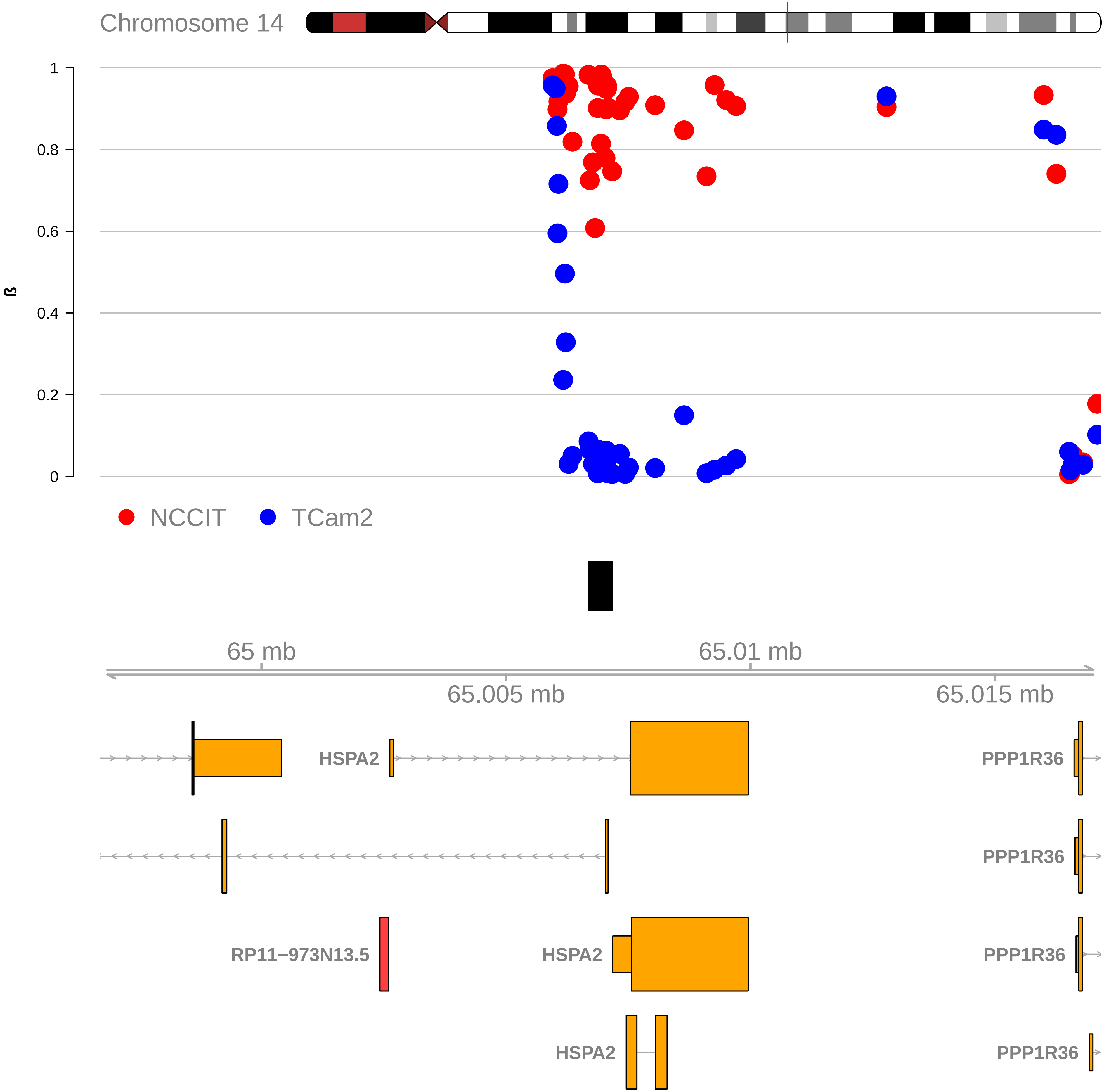

Chromosome 14
1
0.8
0.6
0.4
0.2
0
ß
NCCIT
TCam2
65 mb
65.01 mb
65.005 mb
65.015 mb
HSPA2
PPP1R36
PPP1R36
RP11−973N13.5
HSPA2
PPP1R36
HSPA2
PPP1R36
